# Supplementary figures and images for: The Genome-Wide Analysis of RALF-Like Genes in Strawberry (Wild and Cultivated) and Five Other Plant Species (Rosaceae)
Source: Genes (Basel). 2020 Feb 6;11(2):174. doi: 10.3390/genes11020174 (PMC7073784; doi:10.3390/genes11020174)

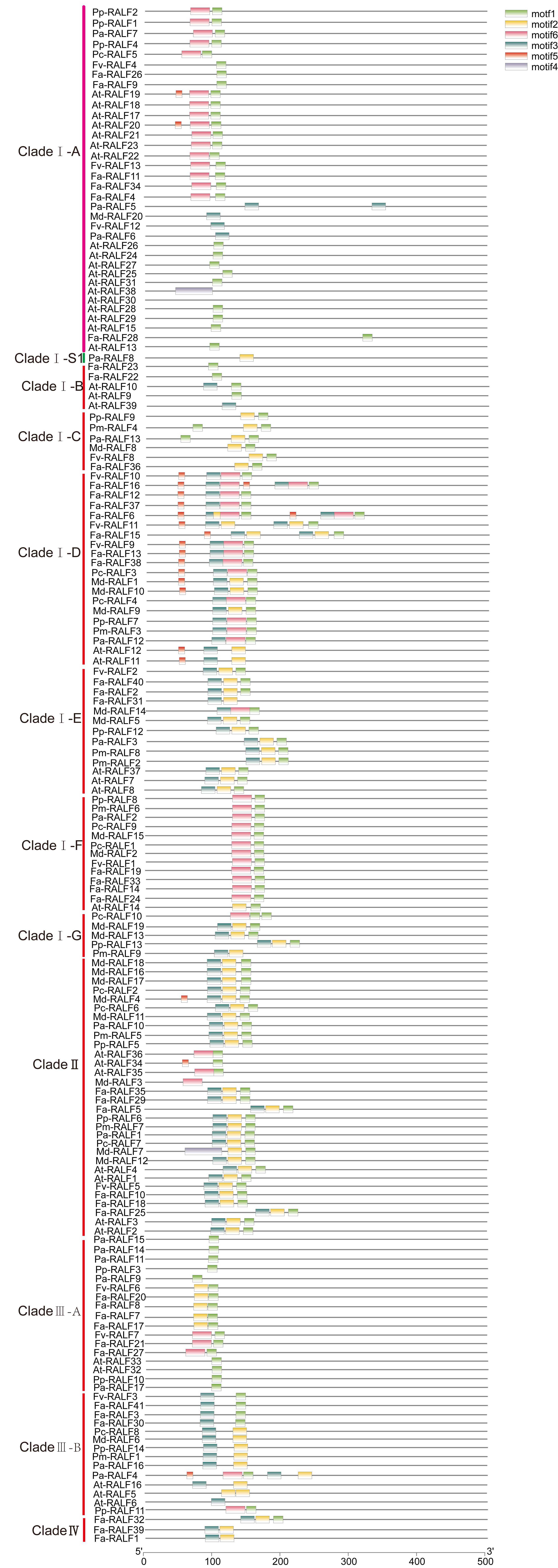

Supplement: Supplementary file 1 [file genes-11-00174-s001.zip › Supplementary Files/Fig S2.tif]

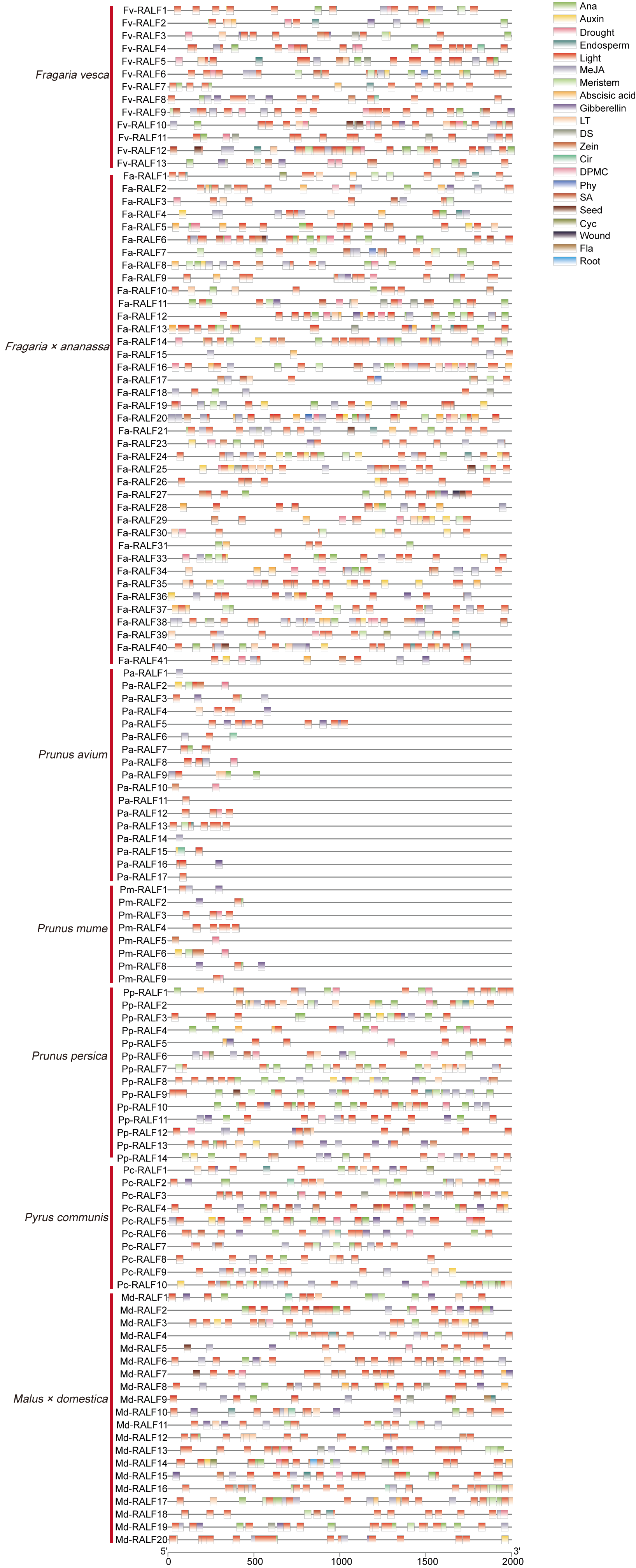

Supplement: Supplementary file 1 [file genes-11-00174-s001.zip › Supplementary Files/Fig S4.tif]

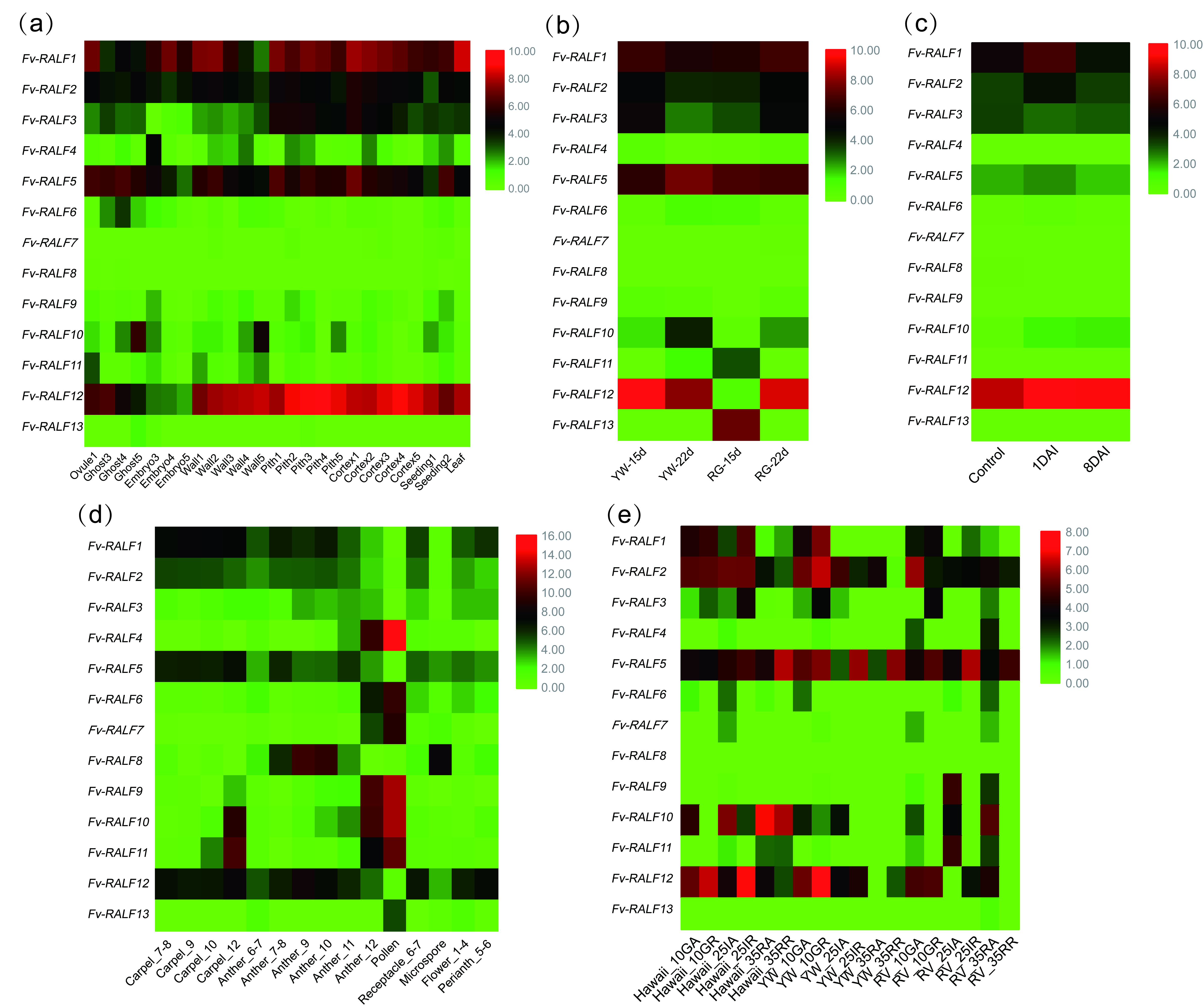

Supplement: Supplementary file 1 [file genes-11-00174-s001.zip › Supplementary Files/Fig S5.tif]

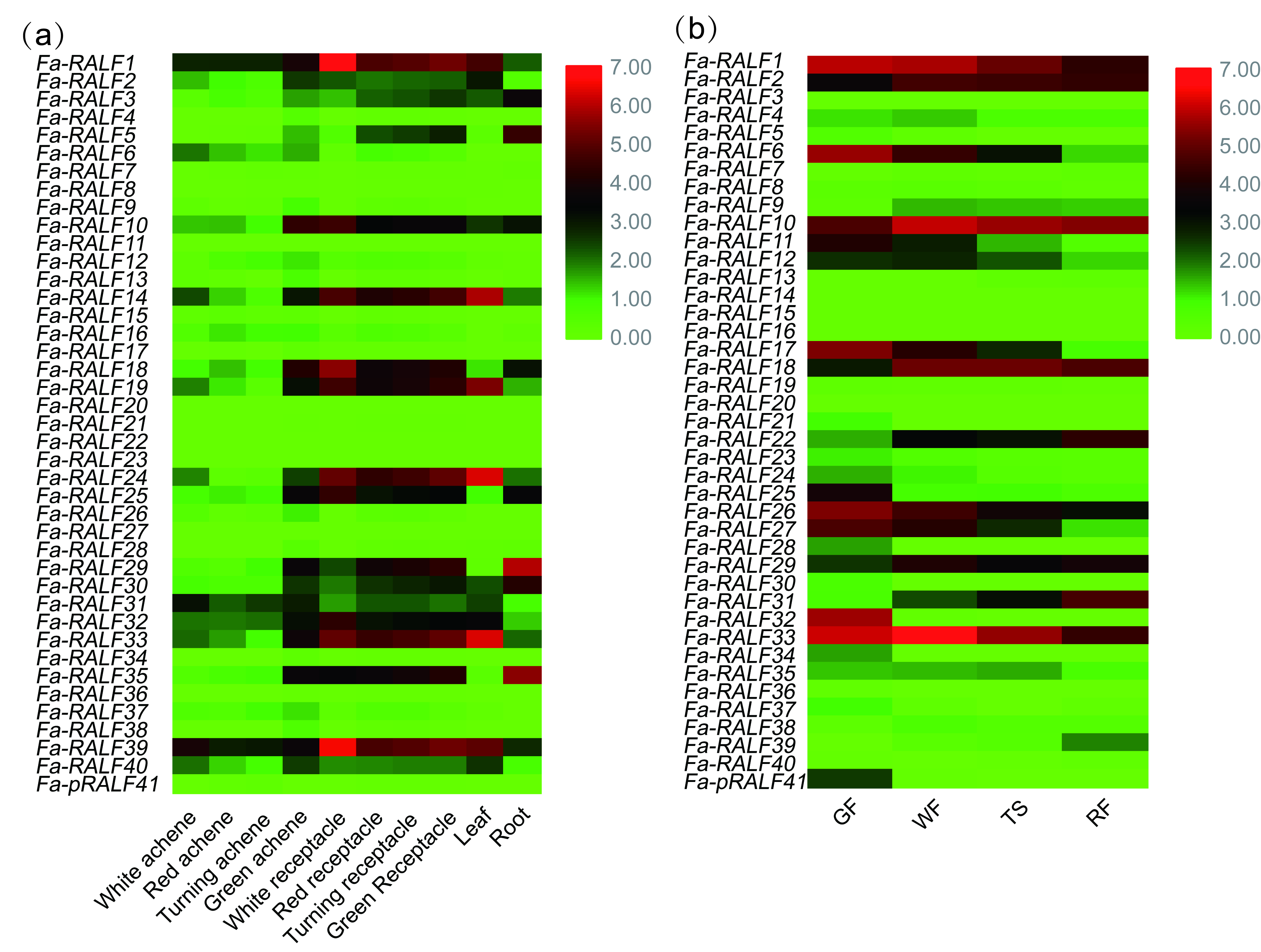

Supplement: Supplementary file 1 [file genes-11-00174-s001.zip › Supplementary Files/Fig S6.tif]

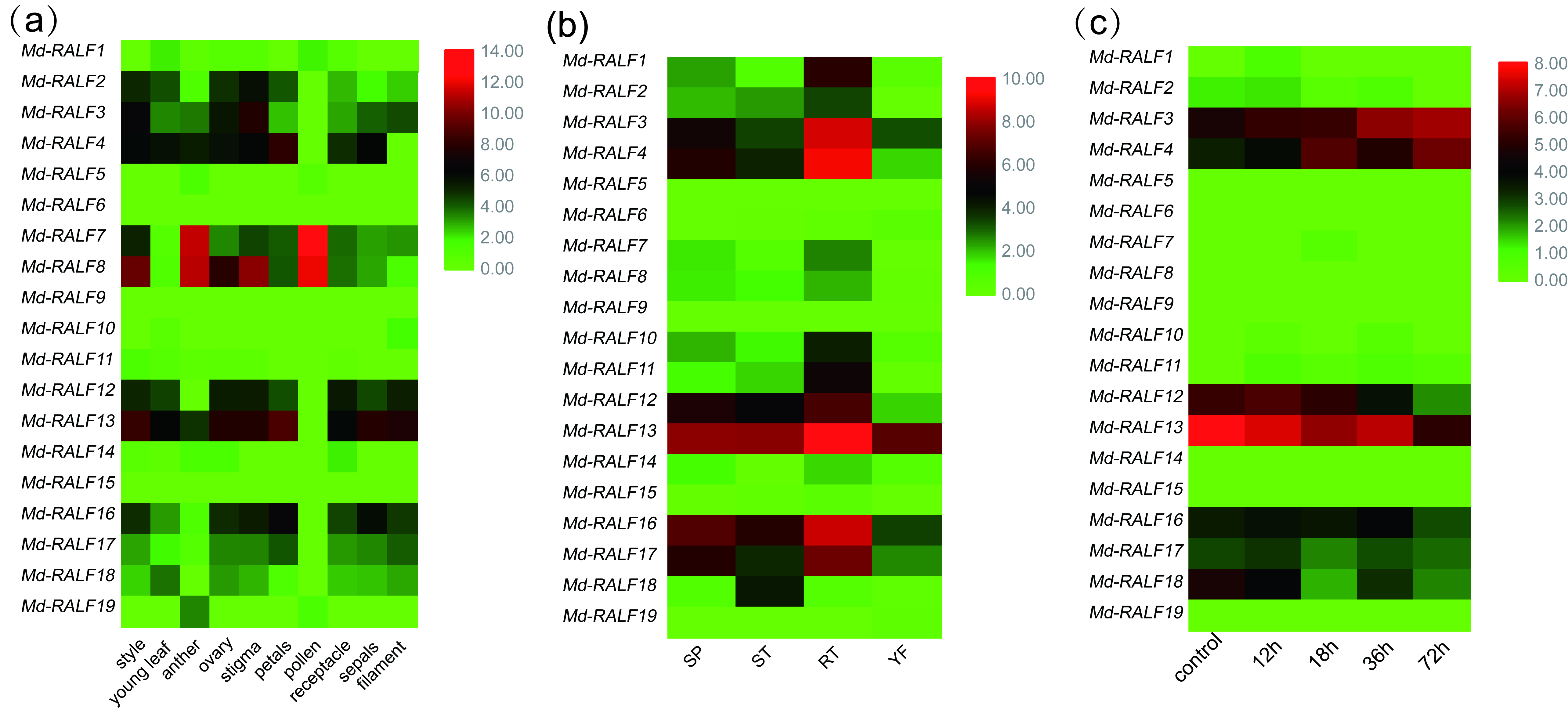

Supplement: Supplementary file 1 [file genes-11-00174-s001.zip › Supplementary Files/Fig S7.tif]
